# Gastric Emphysema in a Critically Ill Patient Successfully Treated without Surgery

**DOI:** 10.1155/2019/1824101

**Published:** 2019-03-18

**Authors:** Hiromi Ihoriya, Tetsuya Yumoto, Masaya Iwamuro, Noritomo Fujisaki, Takaaki Osako, Hiromichi Naito, Atsunori Nakao

**Affiliations:** ^1^Advanced Emergency and Critical Care Medical Center, Okayama University Hospital, Japan; ^2^Department of Gastroenterology and Hepatology, Okayama University Graduate School of Medicine, Dentistry, and Pharmaceutical Sciences, Japan

## Abstract

Gastric emphysema is a relatively rare clinical entity caused by injury to the gastric mucosa. A 62-year-old Japanese male with a history of heavy alcohol consumption and smoking was admitted to the emergency intensive care unit due to severe hypercapnic respiratory acidosis. His body mass index was only 12.6. Ten days after initiation of enteral feeding, he complained of abdominal pain. Computed tomography revealed intraluminal air in the distended gastric wall. Esophagogastroduodenoscopy showed diffuse edema, redness, and erosion throughout the stomach. Based on the findings of narrow angle and short distance of the aorta-superior mesenteric artery, the patient was diagnosed with gastric emphysema associated with superior mesenteric artery syndrome. He was successfully managed nonoperatively with treatments including intravenous antibiotics, gastric decompression, and bowel rest. Physicians should be aware of this unusual condition in such critically ill patients complaining of abdominal pain and needing close monitoring and observation to exclude gastric necrosis or perforation.

## 1. Introduction

Gastric emphysema, which is characterized by the accumulation of gas within the wall of the stomach with or without concomitant portal venous gas, is a relatively rare clinical entity. It can develop secondary to mechanical injury of the gastric surface resulting from elevation of intraluminal pressure such as malignant obstruction of the stomach outlet, superior mesenteric artery syndrome (SMAS), or excessive vomiting [1–5]. Although gastric emphysema is usually self-limiting and resolves spontaneously, it can be challenging to differentiate from emphysematous gastritis and mortality from the condition remains high [6]. To our knowledge, gastric emphysema has never been reported in a critically ill patient. Herein, we describe a rare case of gastric emphysema that might have been induced by SMAS in a patient with malnutrition and alcoholism. This paper should help raise physician awareness of this potentially critical condition.

## 2. Case Presentation

**Figure 1:**
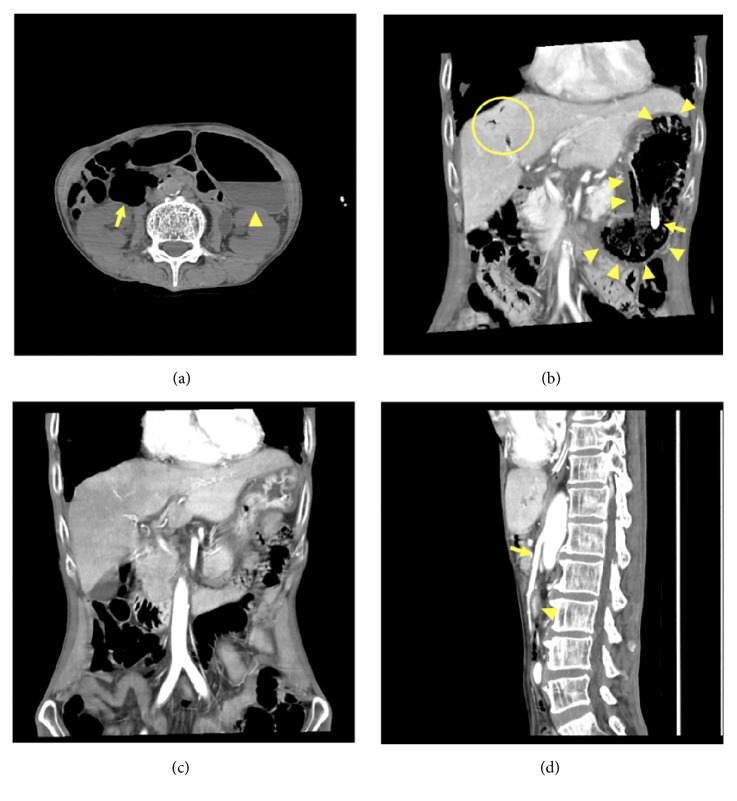
Plain abdominal CT scan on admission showing dilatation of the stomach (arrowhead) and second portion of the duodenum (arrow) (a). Contrast-enhanced CT scan on hospital day 11 showing gas within the wall of a distended stomach (arrowheads) and portal vein (circled region). An arrow demonstrating the tip of the feeding tube (b). Follow-up CT scan showing improvement of gas in the stomach and portal vein (c). Sagittal view showing an acute angle of the aorto-superior mesenteric artery (arrow) and a collapsed duodenum (arrowhead) (d).

A 62-year-old Japanese male with a history of heavy alcohol consumption and smoking was brought to the emergency department due to altered mental status. He was confused and disoriented, with a Glasgow Coma Scale score of 8 (E2V2M4). His vital signs were as follows: respiratory rate: 36 breaths/min; pulse rate: 124 beats/min, blood pressure: 122/84 mmHg; and temperature: 35.9°C. Physical examination was unremarkable except for gross emaciation (height: 160 cm; weight: 32.2 kg; body mass index: 12.6). Also, no obvious abnormal neurological findings including paralysis or ocular movement disorder were observed. Arterial blood gas analysis revealed severe respiratory acidosis with pH: 7.187; PaCO_2_: 110.3 mmHg; PaO_2_: 145.9 mmHg; HCO3^−^: 30.6 mmol/L; base excess: 6.8 mmol/L; lactate: 4.0 mmol/L; glucose: 104 mg/dl on 10 L/min of oxygen. Laboratory data showed hyponatremia of 117 mEq/L without any other abnormal findings. The patient was intubated and mechanically ventilated due to his altered level of consciousness resulting from severe hypercapnic respiratory failure. While computed tomography (CT) examination of the head revealed no abnormalities, abdominal CT showed dilatation of the stomach and second portion of duodenum (Figure 1(a)). He was admitted to the emergency intensive care unit for further management.

The patient received intravenous omeprazole for stress ulcer prophylaxis and ampicillin/sulbactam for suspected aspiration pneumonia from day 1. When his estimated original PaCO_2_ level was restored (around 60 mmHg), his neurological state improved. On the second day, an enteral feeding was initiated through a nasogastric tube at 10 mL/h and advanced by 5 mL/day every 48 hours, as no gastric contents had been drained. Tracheostomy was performed on day 5 because prolonged ventilatory support had been expected.

**Figure 2:**
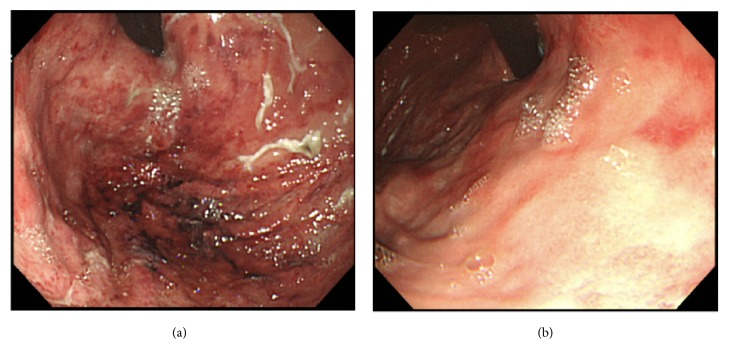
Esophagogastroduodenoscopy demonstrating diffuse mucosal edema, redness, and erosive lesions with white coating throughout the stomach (a), which was resolved (b).

On day 11, he complained of epigastric and left upper quadrant tenderness without guarding or rebound tenderness. In addition, a total of 500 cc of gastric fluid mixed with a material resembling coffee-grounds was drained through the nasogastric tube. His white blood cell count rose to 22,130 /*μ*L with neutrophils 95.8%. Persistent abdominal pain and detection of ascites by ultrasound led us to perform contrast-enhanced CT, which revealed intraluminal air in the distended gastric wall with hepatic portal venous gas (Figure 1(b)). Esophagogastroduodenoscopy (EGD) demonstrated edema, redness, and erosion, with a white coating throughout the stomach without evidence of active bleeding or obvious necrotic lesions (Figure 2(a)).

As the patient had been hemodynamically stable with benign abdominal findings and without elevation of lactate levels, he was successfully managed with nonoperative treatments including intravenous vancomycin and meropenem, bowel rest, and nasogastric tube decompression. Blood cultures obtained on day 11 showed no growth. Follow-up abdominal CT scan on day 19 showed improvement of the gas in the stomach and portal vein (Figure 1(c)). The aorta-superior mesenteric artery (SMA) angle and distance were 17 degrees and 4 mm, respectively (Figure 1(d)), suggesting SMAS as an underlying condition. Complete resolution of the erosive lesions was confirmed on repeat EGD on the same day (Figure 2(b)). Enteral nutrition was resumed uneventfully. Thereafter, the patient was transferred to another hospital for rehabilitation.

## 3. Discussion

Our case highlights the need to consider gastric emphysema, which might have been caused by SMAS, in a critically ill and malnourished patient complaining of abdominal pain. Conservative treatment was effective in treating gastric emphysema in patients with stable hemodynamic status without evidence of gastric necrosis or perforation.

Gastric emphysema is considered a relatively benign condition characterized by intraluminal gas in the stomach resulting from mucosal trauma. It is important to differentiate between gastric emphysema and emphysematous gastritis, both of which exhibit similar radiological findings of gas in the stomach wall with or without portal venous gas [6, 7]. Emphysematous gastritis is a distinct clinical entity related to infection of the gastric wall by gas-forming organisms [8]. The proposed predisposing factors for gastric emphysema resulting from mucosal trauma include gastrointestinal malignancy, gastric volvulus, SMAS, repetitive vomiting, or even nasogastric tube placement [1–7, 9], while immunosuppression, diabetes mellitus, ingestion of corrosives, alcoholism, or nonsteroidal anti-inflammatory drugs have been reported as common underlying conditions in a patient with emphysematous gastritis [6, 8, 10]. Surgical intervention may be warranted in patients with emphysematous gastritis due to development of necrosis or perforation of the stomach [8, 11]. In addition to proper hemodynamic monitoring and management and intravenous antibiotics, EGD should be performed to exclude ischemic stomach [6]. In the present case, benign abdominal findings with hemodynamic stability as well as without evidence of obvious necrotic lesions based on CT examination allowed us to treat the patient with conservative methods including broad-spectrum antibiotics, gastric decompression, and bowel rest.

SMAS is an unusual digestive condition characterized by compression of the third portion of the duodenum due to narrowing of the aorta-SMA angle as a result of extreme weight loss [12]. This clinical entity may cause gastric emphysema, which would be attributed to the elevation of intragastric pressure resulting from intestinal obstruction. To date, only a few cases of gastric emphysema associated with SMAS have been reported [3, 13–15]. Our patient was compatible with SMAS based on the findings of aorta-SMA angle and distance of below 25 degrees and 8 mm, respectively [16]. In addition to SMAS, underlying malnutrition and/or nonocclusive mesenteric ischemia might have contributed to the development of gastric emphysema in the present case [17–19].

As such, this was the first report of gastric emphysema that might have been caused by SMAS in a critically ill and malnourished patient admitted to the intensive care unit due to severe hypercapnic respiratory failure. Critical care physicians should be aware of this clinical entity in such severely ill patients complaining of abdominal pain. Careful hemodynamic monitoring and repetitive physical examination are necessary to exclude gastric necrosis or perforation, which need surgical intervention. Early diagnosis and proper management allow for successful nonoperative management in patients with gastric emphysema.
